# Anticipating EGFR Targeting in Early Stages of Lung Cancer: Leave No Stone Unturned

**DOI:** 10.3390/cells10102685

**Published:** 2021-10-07

**Authors:** Lorenzo Belluomini, Silvia Teresa Riva, Michele Simbolo, Riccardo Nocini, Ilaria Trestini, Alice Avancini, Daniela Tregnago, Miriam Grazia Ferrara, Alberto Caldart, Alessandra Dodi, Anna Caliò, Emilio Bria, Aldo Scarpa, Michele Milella, Jessica Menis, Sara Pilotto

**Affiliations:** 1 Section of Oncology, Department of Medicine, University of Verona Hospital Trust, 37134 Verona, Italy; lorenzo.belluomini08@gmail.com (L.B.); silviateresariva@gmail.com (S.T.R.); ilariatrestini92@gmail.com (I.T.); alice.avancini@univr.it (A.A.); danielatregnago@libero.it (D.T.); alberto.caldart@gmail.com (A.C.); alessandra.dodi92@gmail.com (A.D.); michele.milella@univr.it (M.M.); jessica.menis@aovr.veneto.it (J.M.); 2 Section of Pathology, Department of Diagnostics and Public Health, University of Verona, 37134 Verona, Italy; michele.simbolo@univr.it (M.S.); anna.calio@univr.it (A.C.); aldo.scarpa@univr.it (A.S.); 3 Otolaryngology—Head and Neck Surgery Department, University of Verona Hospital Trust, 37126 Verona, Italy; riccardo.nocini@gmail.com; 4 Comprehensive Cancer Center, Fondazione Policlinico Universitario Agostino Gemelli IRCCS, 00100 Rome, Italy; miriamgraziaferrara@gmail.com (M.G.F.); emilio.bria@unicatt.it (E.B.); 5 Department of Translational Medicine and Surgery, Università Cattolica del Sacro Cuore, 00100 Rome, Italy

**Keywords:** NSCLC, EGFR, early stage, early profiling, ADAURA

## Abstract

*Background*: The current treatment landscape of early stage lung cancer is rapidly evolving, particularly in *EGFR* mutant non-small cell lung cancer (NSCLC), where target therapy is moving to early stages. In the current review, we collected the available data exploring the impact of *EGFR* targeting in both neoadjuvant and adjuvant settings, underlying *lights and shadows* and discussing the existing open issues. *Methods*: We performed a comprehensive search using PubMed and the proceedings of major international meetings to identify neoadjuvant/adjuvant trials with *EGFR* tyrosine kinase inhibitors (TKIs) in NSCLC. *Results*: Limited data are available so far about the activity/efficacy of neoadjuvant TKIs in *EGFR* mutant NSCLC, with only modest downstaging and pathological complete response rates reported. Differently, the ADAURA trial already proposed osimertinib as a potential new standard of care in resected NSCLC harboring an activating *EGFR* mutation. *Conclusion*: Anticipating targeted therapy to early stage *EGFR* mutant NSCLC presents great opportunities but also meaningful challenges in the current therapeutic/diagnostic pathway of lung cancer care. Appropriate endpoint(s) selection for clinical trials, disease progression management, patients’ and treatment selection, as well as need to address the feasibility of molecular profiling anticipation, represent crucial issues to face before innovation can move to early stages.

## 1. Introduction

Lung cancer is the leading cause of cancer-related mortality worldwide, and more than 1.5 million deaths are related to lung cancer every year [1]. The majority of lung cancer patients present with locally advanced or metastatic disease, while only a third of non-small cell lung cancers (NSCLC) is diagnosed in an early resectable stage [2]. Epidermal Growth Factor Receptor (*EGFR*) sensitizing mutations occurring in exon 18–21, firstly described in NSCLC in 2004 [3], were reported with higher prevalence in the Asian population (40–60%) than the Caucasian one (10–20%), as well as in female, younger, and never or light smokers [4]. The discovery of the oncogenic role of EGFR and the consequent development of EGFR tyrosine kinase inhibitors (TKIs), such as erlotinib, gefitinib, afatinib, and osimertinib, revolutionized the disease history of NSCLC, markedly increasing progression-free survival (PFS) in advanced/metastatic settings [5,6,7,8,9]. More recently, the FLAURA trial established osimertinib, a third-generation TKI, as a standard of care in the upfront setting of metastatic NSCLC harboring classical EGFR mutations [10]. As it happened previously for the FLAURA trial, the recent results of the ADAURA study shook the lung cancer oncological scientific community, potentially leading to the anticipation of osimertinib in the adjuvant setting [11]. Thus, while the molecular profiling of NSCLC (including the *EGFR* testing) is considered a standard of care in the advanced setting, the utility and necessity of molecular testing in early stage NSCLC are currently coming under the spotlight. The purpose of this review is to summarize the available data about EGFR TKIs in early stages NSCLC and the rationale supporting early disease profiling. In particular, we aim to explore the controversies beneath these trials in order to discuss if the theory of the best first will be (again) the best choice.

## 2. Targeting EGFR in the Neoadjuvant Setting

Although not so extensively evaluated as in the adjuvant setting, neoadjuvant-platinum-based chemotherapy in NSCLC showed a similar survival outcome, and it might be applied to try to achieve a disease downstaging, potentially leading to a less extensive thoracic cancer surgery. In locally advanced, single-station N2 disease, pre-operatively diagnosed, three different strategies (surgery upfront followed by adjuvant chemotherapy, preoperative chemotherapy followed by surgery, or chemotherapy plus radiotherapy) should be discussed within an experienced multidisciplinary team to define the best patient-tailored approach [12]. A metanalysis published in 2014 demonstrated that a preoperative approach with chemotherapy in early stage NSCLC (I–IIIA) translated into improved survival outcomes, in particular in terms of overall survival (OS-HR 0.87, 95% CI 0.78–0.96; *p =* 0.007), recurrence-free survival (RFS-HR 0.85, 95% CI 0.76–0.94; *p* = 0.002) and in the relative risk of death (13% of reduction) [13].

Despite the fact that EGFR-TKIs in advanced disease did not allow achieving a curative intent so far, the benefit observed in overall response rate (ORR), PFS, and OS set the stage for a promising activity of these small molecules in *EGFR* mutant early stage NSCLC, including the neoadjuvant setting. As a matter of fact, in 2018, a report of two anecdotical cases was published, showing the activity of afatinib in downstaging two locally advanced NSCLC harboring an *EGFR* mutation [14]. Beyond this preliminary observation, the results of a series of phase II trials are available in the neoadjuvant setting of *EGFR* mutant lung cancer [15,16,17,18,19] (Table 1).

In early 2021, a pooled analysis of five phase II prospective clinical trials included 124 patients (mainly Asiatic) affected by stage I–IIIA *EGFR* mutant NSCLC who underwent neoadjuvant erlotinib or gefitinib treatment. The median duration of induction treatment was 42 days (range 21–56 days), and the median time of response evaluation was 45 days (range 42–56 days). Pooled ORR was 58.5% (95% CI 45.5–71.8%). The majority of patients (79.9%) underwent surgical resection; complete resection (R0) was achieved in 64.3% of cases. It is interesting to notice that only modest downstaging and pathological complete response rates were observed, leaving important open questions about the potentiality of neoadjuvant EGFR TKIs to be further explored in the context of phase III clinical trials [29].

Among the available phase II trials, the EMERGING-CTONG 1103 randomized erlotinib vs. gemcitabine plus cisplatin (GC chemotherapy) as neoadjuvant therapy in stage IIIAN2 NSCLC harboring an *EGFR* mutation in exon 19 or 21. Patients received erlotinib 150 mg/d (neoadjuvant therapy, 42 days; adjuvant therapy, up to 12 months) or GC chemotherapy (neoadjuvant therapy, two cycles; adjuvant therapy, up to two cycles). The primary end point of the study was not met: the ORR for neoadjuvant erlotinib versus GC chemotherapy was 54.1% versus 34.3% (odds ratio, 2.26, 95% CI, 0.87–5.84; *p =* 0.092). Three (9.7%) out of thirty-one patients and zero out of twenty-three patients in the erlotinib and GC chemotherapy arms, respectively, had a major pathologic response. No complete pathologic responses were observed. Median PFS was significantly longer with erlotinib (21.5 months) versus GC chemotherapy (11.4 months; HR 0.39; 95% CI, 0.23–0.67; *p* < 0.001) [15]. The final OS analysis was presented at the American Society of Clinical Oncology (ASCO) meeting 2021: median OS was 42.2 months in erlotinib and 36.9 months in the chemotherapy group (HR 0.83, 95% CI, 0.47–1.47; *p* = 0.513). Interestingly, the authors showed that the sequencing of treatments, especially of EGFR TKIs, led to the longest OS: in detail, patients receiving subsequent target therapies (*n* = 38) demonstrated a median OS of 45.8 months, compared to those receiving other (median OS 34.6 months, *n* = 12) or no subsequent treatments (median OS 24.6 months, *n* = 9), with the longest OS in the subgroup of patients in the erlotinib arm further treated with a subsequent TKI (median OS 46.4 months, *n* = 15). Moreover, in this last subgroup of patients undergoing an EGFR TKI rechallenge, ORR was 53.3%, DCR 93.3%, median PFS 10.9 months, supporting its feasibility and activity [20].

After the results of the ADAURA trial [10], a new scenario opened for investigating third-generation EGFR TKIs as induction preoperative therapy. Preliminary data from a phase II study (NCT03433469) indicated that neoadjuvant osimertinib (osimertinib 80 mg orally daily for 1–2 months), in surgically resectable *EGFR* mutant NSCLC, induced pathological responses and downstaging of disease prior to surgery, with a good safety profile [30]. During the International Association for the Study of Lung Cancer (IASLC) 2020 World Conference on Lung Cancer, *Tsuboi* et al. presented the design of NeoADAURA, a phase III, randomized, multicenter study of neoadjuvant osimertinib in *EGFR* mutant resectable stage II‒IIIB NSCLC. The NeoADAURA study will compare the efficacy and safety of neoadjuvant osimertinib as monotherapy or in combination with platinum-based chemotherapy versus chemotherapy alone. Patients will be randomized 1:1:1 to receive either osimertinib at a daily dose of 80 mg or placebo plus the investigator’s choice of pemetrexed at 500 mg/m^2^ plus carboplatin AUC5 mg/mL/min or cisplatin at 75 mg/m^2^ given in 3 cycles every 3 weeks; the third arm of treatment will receive only osimertinib at a daily dose of 80 mg for 9 weeks. The primary end point of the study is centrally assessed major pathological response (MPR), which is defined as less than or equal to 10% of residual cancer cells in the lung tumor specimen after surgery. The secondary endpoints are event-free survival, complete pathological response, downstaging, disease-free survival (DFS), OS, MPR in patients with or without detectable *EGFR* mutations in plasma circulating tumor DNA at screening, disease-related symptoms, and health-related quality of life, as well as safety and tolerability. After surgery, osimertinib will be offered to all patients (eventually adjuvant chemotherapy according to the investigator’s choice) for up to 3 years or until disease recurrence [31] (Table 2).

Overall, the neoadjuvant context of lung cancer represents a critical moment where different issues should be simultaneously addressed. First of all, the limited data available so far do not allow us to drawn definitive conclusions about the activity/efficacy of EGFR TKIs and the results of the ongoing trials, as the NeoADAURA will probably help to clarify their real impact. Second, (*i)* considering that it is a strategy with a particular indication in lung cancer, the selection of patients who could benefit more from a neoadjuvant approach, (*ii)* the optimal duration and interruption time of TKIs before surgery, (*iii)* the best therapeutical strategy (TKI alone or with chemotherapy and potential room for other combinations, as chemo-immunotherapy), and (*iv)* how to manage disease progression and tumor evolution are not yet established. Finally, the molecular profiling of lung cancer in the neoadjuvant setting may be particularly challenging due to the usually scarce tissue available for the analysis, the limited timing for obtaining the results of tumor profiling, as well as to the important coordination and alignment required by the lung cancer multidisciplinary team.

## 3. Targeting EGFR in the Adjuvant Setting

Adjuvant cisplatin-based chemotherapy should be offered to patients with resected stage II and III NSCLC and can be considered in resected stage IB disease and a primary tumor> 4 cm [12]. Like in other solid tumors, the adjuvant systemic approach, both chemotherapy- or targeted-therapy-based (i.e., in melanoma), aims to treat and control the micrometastatic radiographically invisible disease, preventing distant spread, and therefore improving the cure rate. A meta-analysis from five randomized trials, including 4584 NSCLC patients, reported a modest benefit (5.4% absolute 5-year OS benefit) with the use of adjuvant chemotherapy [32]. Indeed, the choice of administering adjuvant chemotherapy should be made on a case-by-case basis, carefully considering clinical and pathological variables of the resected disease (such as stage, performance status, age, and comorbidities). Historically, OS is considered the optimal endpoint to definitely assess the efficacy of adjuvant treatments, and DFS has been validated as a surrogate endpoint of OS in a large metanalysis including 7626 NSCLC patients conducted by Maugen et al., which reported a strong association between DFS and OS in patients treated with adjuvant chemotherapy with or without radiotherapy [33]. Although this association has not (yet) been validated with immunotherapy or targeted agents, in recent years, DFS was frequently applied as the primary endpoint in randomized trials involving *EGFR*-mutated resected NSCLC treated with EGFR TKIs.

Following successful experience in the metastatic setting where different EGFR TKIs are currently approved [5,6,7,8,9,10], several studies aimed to demonstrate a survival advantage with the use of these agents as adjuvant therapy in *EGFR*-mutated resected NSCLC (Table 1). The BR19 phase-three study randomized patients with NSCLC stage IB–IIIA to receive gefitinib or placebo after radical surgical resection. Notably, only 17% of patients received adjuvant chemotherapy, and only 15 patients were known to have an *EGFR* mutation. The trial was closed early on due to safety concerns. At a median follow-up of 4.7 years, there was no difference in OS and DFS between the two treatment arms, including the *EGFR* mutant subgroup (DFS: HR 1.84; 95% CI, 0.44–7.73; *p* = 0.395 and OS: HR 3.16; 95% CI 0.61–16.45; *p* = 0.15) [21]. The RADIANT trial was a randomized phase three trial, including patients with NSCLC stage IB to IIIA expressing EGFR positivity by immunohistochemistry (IHC) or *EGFR* amplification by fluorescence in situ hybridization (FISH). These patients were randomized (2:1) to receive erlotinib vs. placebo for two years. Adjuvant chemotherapy could be administered if indicated. The primary endpoint of the study DFS was not met. In the subgroup of patients harboring *EGFR*-sensitizing mutations (163 out 973 patients enrolled), DFS was superior in the experimental arm (HR 0.61, 95% CI 0.38–0.98; *p* = 0.0391), but, given the hierarchical analysis, this result was not considered statistically significant [22]. The first trial testing the efficacy of erlotinib in *EGFR* mutated radically resected NSCLC was the phase two single-arm SELECT trial. Patients with resected stage IA–IIIA *EGFR* mutant NSCLC, after receiving adjuvant chemotherapy with or without radiation, were enrolled to receive daily erlotinib for two years. The study was designed for 100 patients and powered to demonstrate a primary end point of 2-year DFS greater than 85%, improving the historic data of 76%. Results of this study demonstrated a 2-year DFS of 88%, with a median time to recurrence of 25 months after stopping erlotinib. Interestingly, patients who rechallenged with erlotinib after recurrence (n = 26; 65%) experienced durable benefit (median duration of 13 months) [23]. Afterward, the CTONG1104/ADJUVANT was a phase three trial evaluating the efficacy of adjuvant gefitinib versus standard platinum-based chemotherapy in resected stage II–IIIA *EGFR* mutant NSCLC. Enrolled patients were randomized 1:1 to receive adjuvant gefitinib for 2 years or vinorelbine plus cisplatin for four cycles. The primary endpoint was DFS in the intention-to-treat population, while secondary endpoints were OS, 3 and 5-year DFS rate, and 5-year OS rate. At a median follow-up of 36.5 months, the study demonstrated a significantly increased median DFS among patients receiving gefitinib (28.7 vs. 18.0 months; HR 0.60, 95% CI 0.42–0.87; *p* = 0.0054) [24]. Nevertheless, at a median follow-up of 76.9 months, no difference in median OS was demonstrated (75.5 and 79.2 months, respectively). However, the authors concluded that DFS advantage did not translate into OS difference; an mOS of 75.5 months was the best result compared to historical data [25]. The phase two EVAN trial randomized 102 patients with radically resected stage IIIA *EGFR* mutant NSCLC to receive adjuvant erlotinib for up to two years vs. cisplatin-vinorelbine chemotherapy. The primary endpoint was 2-year DFS. At a median follow-up of 33 months, the 2-year DFS was 81.4% in the experimental arm and 44.6% in the chemotherapy arm (RR 1.82, 95% CI 1.19–2.78; *p* = 0.0054). Despite these results being promising, the small sample size and the immature data for OS do not allow to provide definitive conclusions [26]. In another phase two clinical trial, patients with resected IIIA N2 NSCLC harboring *EGFR* mutations (either exon 19 deletion or L858R point mutation) were assigned to four cycles of carboplatin-pemetrexed, followed or not by gefitinib for 6 months. The primary endpoint DFS was significantly longer among patients who received chemotherapy followed by 6 months gefitinib than those who received only adjuvant chemotherapy (39.8 versus 27.0 months; HR 0.37, 95% CI: 0.16–0.85; *p* = 0.014). Furthermore, this trial demonstrated an advantage in 2-year mOS in the chemotherapy plus gefitinib arm (HR 0.37, 95% CI 0.12–1.11; *p* = 0.076) [27]. Recently at ASCO meeting 2021, the results of a randomized phase three trial, IMPACT, evaluating adjuvant gefitinib versus cisplatin/vinorelbine in Japanese patients with completely resected, *EGFR*-mutated, stage II–III NSCLC, were presented. Overall, 234 patients were randomly assigned to receive either gefitinib for 24 months or cisplatin plus vinorelbine for four cycles. No significant differences were seen in both DFS, the primary endpoint of the study (HR 0.92, 95% CI 0.67–1.28; *p* = 0.63) and OS (HR 1.03; *p* = 0.89). Of note, the most common site of distant metastasis was the brain, occurring in 26 patients in the gefitinib arm and in 14 patients in the chemotherapy arm, respectively. Among 65 patients undergoing treatment after gefitinib relapse, 67% were further treated with TKIs, while 98% of patients in the cisplatin/vinorelbine arm who experienced relapse were subsequently treated with TKIs [28].

Considering these promising data, several clinical trials with EGFR TKIs in the adjuvant setting are currently ongoing ( Table 2 ). Among these trials, the ALCHEMIST-EGFR (NCT02193282), probably the largest effort to address the role of EGFR TKI in the adjuvant setting, is a phase three study evaluating the efficacy of adding erlotinib for 2 years vs. placebo in patients with fully resected *EGFR* mutant stage IB–IIIA NSCLC, with OS as a primary endpoint. Another phase three trial is EVIDENCE (CCTC-1501; NCT02448797), comparing icotinib with standard chemotherapy in *EGFR* mutant stage II–IIIA NSCLC.

### The ADAURA Trial: Lights and Shadows

Osimertinib is an oral medication that is a third-generation TKI, approved—thanks to the results of the FLAURA trial—as first-line treatment in *EGFR*-mutated advanced NSCLC [10,34]. The ADAURA trial was a phase three clinical trial that randomized patients (1:1; random assignment was stratified by stage (IB vs. II vs. IIIA), type of *EGFR* mutation (exon 19 del vs. L858R), and race (Asian vs. non-Asian), no stratification based on adjuvant chemotherapy) with radically resected stage IB–IIIA NSCLC to receive adjuvant osimertinib at the dose of 80 mg/die up to 3 years vs. observation. In this trial, osimertinib was added to the standard of care strategy, including adjuvant chemotherapy, while postoperative radiotherapy (PORT) was not allowed. The study was a superiority trial with DFS as the primary endpoint in stage II–IIIA, while DFS in the intention-to-treat population and OS were among the secondary endpoints. The results of the ADAURA trial were first presented at ASCO 2020 plenary session and then published in the *New England Journal of Medicine* [11]. Overall, 339 patients were assigned to the osimertinib arm and 343 to the placebo arm. Approximately 60% of the patients in each arm received adjuvant chemotherapy. The published results were derived from an unplanned interim analysis at the data cut-off of January 12, 2020. At 24 months, 90% of the patients with stage II–IIIA disease in the osimertinib group and 44% of those in the placebo group were alive and disease-free (HR 0.17, 99.06% CI 0.11–0.26; *p* < 0.001). Additionally, in the overall population, DFS was significantly longer (89% patients in the osimertinib group and 52% in the placebo group were alive and disease-free at 24 months (HR 0.20, 99.12% CI 0.14–0.30; *p* < 0.001)), with a consistent benefit across all the predefined subgroups (age, smoking, race, stage, and type of *EGFR* mutation) and irrespectively of the administration of adjuvant chemotherapy. The magnitude of benefit was greater in stage IIIA (HR 0.12) than in stage IB (HR 0.39). An impressive result was presented about central nervous system (CNS) DFS: CNS recurrence or death occurred in 45 patients (2% in the osimertinib arm vs. 11% in the placebo arm; HR 0.18; 95% CI, 0.10–0.33). No new safety concerns were noticed.

Since the presentation of the ADAURA results, an intense debate has arisen across the thoracic oncology community. Despite the impressive HR for DFS, some limitations of ADAURA have been highlighted.

First of all, the study protocol allowed the enrollment of patients without positron emission tomography-computed tomography (PET-CT) scan and brain MRI at screening, despite these techniques represent the standard of care for staging early stage NSCLC [35]. Thus, lacking an accurate baseline staging, it is not possible the exclude the presence of understated stage IV among the enrolled patients. Second, the safety of osimertinib should be subject to careful long-term monitoring considering the 3-year treatment period and the adjuvant scenario. Moreover, although the benefits of adjuvant chemotherapy are modest, in the ADAURA trial, the HR for 2-year DFS was superior in patients who received adjuvant chemotherapy vs. those who did not (0.16 vs. 0.23), suggesting some incremental benefit of chemotherapy in addition to osimertinib, further confirming that, at least with the evidence available so far, adjuvant chemotherapy remains a standard of care for chemo-eligible patients affected by resected *EGFR* mutant NSCLC, in cases prior to TKIs. Furthermore, OS represents the gold standard endpoint in the adjuvant setting, since, so far, the DFS benefit with EGFR TKIs did not anticipate an OS improvement and DFS has been statistically validated as a surrogate end point for OS with chemotherapy [33], but whether this surrogacy is confirmed when evaluating distinct drugs remains to be clarified. Nevertheless, while waiting for a longer follow-up and the secondary OS endpoint results, the availability of a drug able to delay disease recurrence, prevent CNS metastases (a well-known critical site of relapse also after adjuvant first-generation TKIs [28]), while maintaining the patient’s quality of life, represents an important step forward in the adjuvant approach to *EGFR* mutant NSCLC.

## 4. Targeting EGFR in Locally Advanced NSCLC: A New Horizon?

Based on the results of the PACIFIC trial [36], the current standard of care in stage III NSCLC includes the combination of chemoradiotherapy followed by 1-year of durvalumab. Despite the impressive results of the PACIFIC trial in terms of both PFS and OS, some open issues are still unanswered regarding, for example, the efficacy in the oncogene-addicted population. This subgroup of patients showed an improved PFS (HR 0.76) with durvalumab, although only 43 *EGFR* mutant NSCLC patients were enrolled in the PACIFIC study, and the confidence interval crossed 1 [37]. Thus, further analysis about durvalumab efficacy in the *EGFR* mutant, as well as in the oncogene-addicted population in general, is urgently needed. In this light, recently, Aredo et al. presented the results of a retrospective study involving patients affected by stage III *EGFR* mutant NSCLC treated with chemoradiation with or without durvalumab [38]. The 37 stage III *EGFR* mutant NSCLC patients underwent different treatment approaches (chemoradiation alone (*n* = 16); chemoradiation followed by consolidative durvalumab (*n* = 13); induction TKI followed by chemoradiation (*n* = 4); and chemoradiation followed by TKI (*n* = 4)), highlighting how heterogenous the management of this subpopulation is in clinical practice. The 13 patients treated with consolidative durvalumab had a PFS that was not significantly different from patients treated with chemoradiation alone (10.3 vs. 6.9 months; *p* = 0.993), with a particularly relevant toxicity profile, considering that around 50% of patients developed a severe adverse event (25% pneumonitis). The limited efficacy of immunotherapy single-agent in *EGFR* mutant NSCLC was already recognized in the advanced setting [39], probably related to the low T-cell infiltration and tumor mutational burden and the intrinsic lack of immunogenicity of the oncogene-addicted disease [40]. Considering this, even though no definitive conclusions can be drawn about the effective role of immunotherapy in stage III *EGFR* mutant NSCLC, prospective trials in this population are urgently needed. In this regard, at the IASLC 2021 Targeted Therapies of Lung Cancer Meeting, the final results of the ASCENT trial were presented. This trial aimed to evaluate the integration of afatinib into standard-of-care chemoradiation with or without surgery in stage III *EGFR* mutant NSCLC. Patients received afatinib 40 mg once a day for 2 months and then underwent restaging based on the primary end-point assessment (ORR). Afterwards, patients received chemoradiation (radiation therapy plus concurrent four cycles of cisplatin-pemetrexed) or induction chemoradiation before surgical resection. If there was no evidence of progressive disease, patients received 2 years afatinib as adjuvant treatment. The planned sample size was 30 patients, however the study closed for slow accrual in 2020. In the 19 enrolled patients, ORR after 2 months of neoadjuvant afatinib was 58%. Among nine patients with an unresectable disease who had completed neoadjuvant therapy, one patient progressed, one patient converted to operable, and seven patients proceeded to definitive chemoradiotherapy. The median PFS was 34.6 months, and the median OS was 69.1 months [41]. Besides the ASCENT study, the LAURA trial (NCT03521154) is currently evaluating the efficacy of osimertinib as a consolidative treatment after chemoradiation in patients with unresectable stage III *EGFR* mutant NSCLC.

## 5. Open Issues: Managing Progression, Patient’s Selection, and Profiling Anticipation

**Figure 1:**
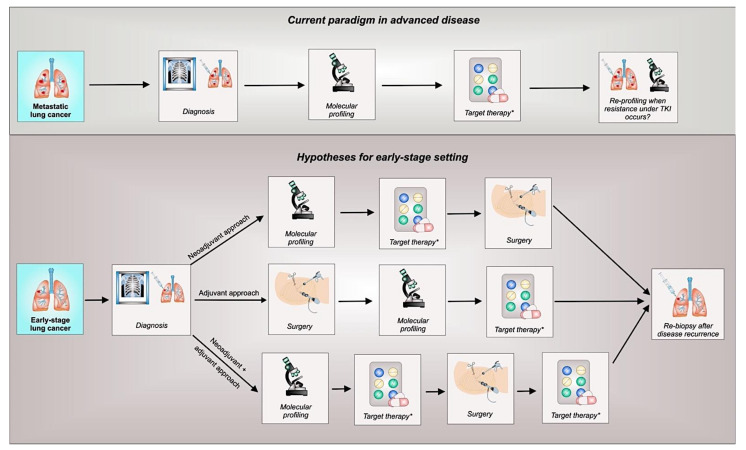
Current paradigm in advanced NSCLC decision-making process and hypotheses for early stage settings. Legend: TKI, tyrosine kinase inhibitor. * Standard of care (i.e., chemotherapy) in the different settings is to be considered according to clinical trials results or current guidelines.

The early introduction of targeted therapy in the disease trajectory of *EGFR* mutant NSCLC, as well as other oncogene-addicted diseases, holds great opportunities but also meaningful challenges (Figure 1).

Probably the most investigated question, when TKIs are anticipated to the adjuvant setting for oncogene-addicted disease, is about the management of disease progression in case of relapse. In this regard, the recent results of EMERGING-CTONG 1103 and IMPACT trials suggested that if patients are not definitely cured by EGFR TKIs in the early disease setting, rechallenge is possible with an expected good possibility of persistent activity and prolonged survival benefit [20,28]. Moreover, lessons we are learning in the advanced setting may be useful for customizing the therapeutical approach according to the molecular background of the disease and its evolution over time. Although many open issues are still to be answered regarding the management of tumor heterogeneity/resistance and, in this sense, the potential room for treatment combinations at baseline or at progression [42], innovative diagnostic tools coming from the preclinical as patient-derived organoids (PDOs) may accelerate the identification and development of effective therapeutic strategies able to assist the clinical decision-making process [43]. In this light, the identification and validation of reliable predictive biomarkers represent a crucial gap to be fulfilled.

Another important strategy in the adjuvant setting is based on the optimization in patient selection. Although a tailored adjuvant chemotherapy approach did not lead so far to a survival benefit in patients with completely resected NSCLC, as observed in the phase III ITACA trial [44], the presence of an oncogenic alteration may specifically drive the benefit of adjuvant TKIs. Further crucial information may come from the wider application of next-generation sequencing technologies for liquid biopsy testing in NSCLC [45]. In this regard, although methodological challenges related to the low ctDNA concentration into the bloodstream in early disease stages, a liquid biopsy may help in patient’s selection for both neoadjuvant therapy, considering that pre-treatment ctDNA is prognostic, and adjuvant treatment, because the persistence of minimal residual disease (MRD) has been associated with an increased risk of disease recurrence, potentially allows the personalization of an adjuvant/consolidation approach [46]. Of interest, new generation studies are applying liquid biopsy tools to identify and select those patients at higher risk of disease recurrence for an escalation of care in the adjuvant setting [47].

Finally, after the presentation of the ADAURA trial results, the thoracic oncology community has started to speculate about the potential utility and feasibility of anticipating tumor profiling in early disease settings (Figure 1). About the clinical utility of comprehensive genomic profiling, although data are available in the metastatic setting, several potential advantages have been reported, including the increased probability of obtaining successful samples and sensitivity in actionable targets detection, leading to a potential improvement in clinical outcomes [48]. Nevertheless, when dealing with the real-world feasibility of molecular testing in an advanced setting, a retrospective observational chart review study including almost 3500 NSCLC patients initiating first-line systemic therapy between 2018 and 2020 in several US centers reported that <50% of them received all five tests that are necessary for a recommended first-line treatment program, and that NGS testing was accessible in <50% of cases [49]. This probably mirrors a widespread criticism related to NGS implementation in lung cancer, which is still lacking dedicated economical and staff resources.

In conclusion, lung cancer care is evolving almost day by day thanks to the increased understanding of disease biology, the availability of molecular profiling, as well as the development of new therapeutical weapons. In this light, the anticipation of this innovation in early disease stages represents a unique opportunity to definitively change the prognosis of our patients, and a strong effort from all the scientific community should be performed in order to accelerate the transition from theory to our everyday clinical practice.

## Figures and Tables

**Table 1:** Main neoadjuvant and adjuvant clinical trials testing EGFR TKIs in NSCLC.

*Trial*	*Ph*	*Stage*	*Study Arm(s)*	*N*	*Primary Endpoint*	*Main Results*	*Safety* *(AEs Grade 3–4)*
***Neoadjuvant***							
*EMERGING CTONG 1103* [15,20]	II	IIIA–N2	Erlotinib ChT	37 (E) 35 (ChT)	ORR	- *ORR*: 54% (E) vs. 34% (ChT) - (OR 2.26, 95% CI 0.87–5.84, p = 0.092) - *mOS*: 42.2 mo (E) vs. 36.9 mo (ChT) - (HR 0.83, 95%CI 0.47–1.47, p = 0.513)	0 (E) 29.4% (ChT)
*NCT01217619*^#^ [16]	II	IIIA	Erlotinib ChT	15 (E) 16 (ChT)	Radical resection rate	- *ORR*: 67% (E) vs. 19% (ChT) (NS) - *mDFS*: 10.2 mo (E) vs. 8.0 mo (ChT) (p = 0.25) - *mOS*: 51.0 mo (E) vs. 20.9 mo (ChT) (p = 0.12)	NA
*NCT00600587* * [17]	II	IIIA–N2	Erlotinib ChT	12 (E) 12 (ChT)	ORR	- *ORR*: 58.3% (E) vs. 25.0% (ChT) (p = 0.18) - *mPFS*: 6.9 mo (E) vs. 9.0 mo (ChT) (p = 0.071) - *mOS*: 14.5 mo (E) vs. 28.1 mo (ChT) (p = 0.2)	16.7% (skin rash) (E) *No better specified*
*Rizvi NA, et al*. [18]	II	I–II	Gefitinib	50	Correlation of radiographic response with *EGFR* mut	- *ORR*: 17/21 with an EGFR mutation and 4/21 without (p = 0.0001)	1/50 (diarrhea) (G)
*NCT01833572* [19]	II	II–IIIA	Gefitinib	33	ORR	- *ORR*: 54.5% - *mDFS*: 33.5 mo	0
***Adjuvant***							
*BR.19* ^‡^ [21]	III	IB–IIIA *15 EGFR mutant*	Gefitinib (2 y) Placebo (2 y)	251 (G) 252 (p)	OS DFS	- *DFS*: 4.2 y (G) vs. NR (p) - (HR 1.22, 95% CI 0.93–1.61; p = 0.15) - *OS* : 5.1 y (G) vs. NR (p) - (HR 1.24, 95% CI 0.94–1.64; p = 0.14)	5–8% (mainly rash, diarrhea, dyspnea) (G)
*RADIANT* [22]	III	IB–IIIA *EGFR pos* *(IHC/FISH*)	Erlotinib (2 y) Placebo (2 y)	623 (E) 350 (p)	DFS	- *DFS*: 50.5 mo (E) vs. 48.2 mo (p) - (HR 0.90, 95% CI 0.74–1.10; p = 0.324) - *DFS* in EGFR mut: 46.4 (E) vs. 28.5 (p) - (HR 0.61, 95% CI 0.38–0.98; p = 0.0391)	22.3% (rash) (E) 6.2% (diarrhea) (E)
*SELECT* [23]	II	I–IIIA	Erlotinib (2 y)	100	2 y DFS	- *2 y DFS*: 88% - (vs.historical control 76%; *p* = 0.0047)	13% (rash) (E) 3% (diarrhea) (E)
*CTONG1104/* *ADJUVANT* [24,25]	III	II–IIIA	Gefitinib (2 y) ChT (4 cycles)	222	DFS	- *DFS*: 28.7 mo (G) vs. 18 mo (ChT) - (HR 0.60, 95% CI 0.42–0.87; p = 0.0054) - *mOS* : 75.5 mo (G) vs. 79.2 mo (ChT) - (HR 0.96, 95%CI 0.64–1.43; p = 0.823)	12% (G) 48% (ChT)
*EVAN* [26]	II	IIIA	Erlotinib (2 y) ChT (4 cycles)	51 (E) 51 (ChT)	2 y DFS	- *2 y DFS*: 81.4% (E) vs. 44.6% (ChT) - (RR 1.823, 95% CI 1.194–2.784; p = 0.0054) - *DFS*: 42.4 mo (E) vs. 21 (ChT) - (HR 0.268, 95% CI 0.136–0.531; p < 0.0001)	12% (E) 26% (ChT)
*Li et al*. [27]	II	IIIA N2	ChT (4 cycles) +/− Gefitinib (6 mo)	30 (ChT-G) 30 (ChT)	DFS	- *DFS*: 39.8 mo (ChT-G) vs. 27 mo (ChT) - (HR 0.37, 95% CI 0.16–0.85; p = 0.014) - *2 y OS:* 92.4% (ChT-G) vs. 77.4% (ChT) - (HR 0.37, 95% CI 0.12–1.11; p = 0.076)	20% (ChT-G) 16.7% (ChT)
*ADAURA* [11]	III	IB–IIIA	Osimertinib (3 y) Placebo (3 y)	339 (O) 343 (p)	DFS	- *DFS*: NR (O) vs. 20.4 mo (p) - (HR 0.17, 99.06% CI 0.11–0.26; p < 0.001) - *DFSrate*: 89% (O) vs. 52% (p) - (HR 0.20, 99.12% CI 0.14–0.30; p < 0.001)	20% (diarrhea, stomatitis) (O)
*IMPACT* [28]	III	II–IIIA	Gefitinib (2 y) ChT (4 cycles)	116 (G) 116 (ChT)	5 y DFS	- *DFS*: 35.9 mo (G) vs. 25.0 mo (ChT) - (HR 0.92, 95% CI 0.67–1.28; p = 0.63) - *5 y survival rates*: 78.0% (G) vs. 74.6% (ChT) (HR 1.03, 95% CI 0.65–1.65; p = 0.89)	NA

Legend: N, number; AEs, adverse events; ChT, chemotherapy; ORR, overall response rate; E, erlotinib; G, gefitinib; mOS, median overall survival; mo, months; DFS, disease-free survival; ChTRT, chemoradiotherapy; MPR, major pathological response; mPFS, median progression-free survival; y, years; NR, not reached; O, osimertinib; RR, relative risk; NA, not available; NS, not statistically significant. ^*#*^ Previously published as a single-arm study (*Xiong L., et al. Oncologist 2019; 24: 157-e64*) * Treatment assignment based on *EGFR* mutation status ^‡^ Early closure for safety concerns.

**Table 2:** Ongoing trials with EGFR TKIs in neoadjuvant and adjuvant settings.

*Trial Identifier*	*Phase*	*Main* *Inclusion* *Criteria*	*Study Arm(s)*	*Duration of TKIs*	*Primary Endpoint*	*Status*
***Neoadjuvant***						
*NCT01833572*	II	EGFR mutant (19del/L858R); resectable stage II–IIIA	Gefitinib	42 days before surgery	ORR	Unknown†
*NCT03433469*	II	EGFR mutant (19del/L858R); resectable stage I–IIIA	Osimertinib	1–2 cycles q28 before surgery	MPR	Recruiting
*ChiCTR1800016948*	II	EGFR mutant (19del/L858R); resectable stage II–IIIA	Osimertinib	6 weeks before surgery	ORR	Recruiting
*NCT04351555-* *NeoADAURA*	III	EGFR mutant (19del/L858R, alone or in combination with other mutations, i.e., T790M); resectable stage II–IIIB N2	Cis-carboplatin/pemetrexed vs. cis-carboplatin/pemetrexed + osi vs. osimertinib alone	3 cycles q21 (chemotherapy arms), ≥9 weeks osimertinib	MPR	Recruiting
***Adjuvant***						
*NCT03381066*	III	EGFR mutant (19del/L858R); resected stage IIA–IIIB (excluding N3)	Gefitinib, pemetrexed, cisplatin vs. vinorelbine, cisplatin	1 year	DFS	Recruiting
*NCT02448797*	III	EGFR-mutant; resected stage II–IIIA	Icotinib vs. 4 cycles adjuvant-platinum-based ChT	2 years	DFS	Recruiting
*NCT04687241*	III	EGFR mutant (19del/L858R, alone or in combination with other mutations, i.e., T790M); resected stage IIA–IIIB (only T3N2M0)	Almonertinib vs. placebo	NA	DFS, assessed by IRC	Not yet recruiting
*NCT04853342*	III	EGFR mutant (19del/L858R, alone or in combination with other mutations, i.e., T790M); resected stage IB–IIIA	Furmonertinib (AST2818) vs. placebo	NA	DFS	Not yet recruiting
*NCT02125240*	III	EGFR-mutant, resected stage II–IIIA	Icotinib vs. placebo	NA	DFS	Unknown ^†^
*NCT01996098*	III	EGFR-mutant, resected stage II–IIIA	6 mo icotinib (following ChT) vs. 12 mo icotinib (following ChT) vs. chemotherapy	6 months/12 months	DFS	Recruiting
*NCT04762459*	III	EGFR mutant (19del/L858R, alone or in combination with other mutations, i.e., T790M); resected stage II–IIIA	Almonertinib vs. almonertinib plus pemetrexed plus cisplatin vs. pemetrexed plus cisplatin alone	NA	DFS	Not yet recruiting
*NCT02264210*	II	EGFR-mutant, resected stage IB	Icotinib vs. observation	1 year	OS	Recruiting

Legend: ORR, overall response rate; MPR, major pathological response; DFS, disease-free survival; IRC, independent review committee; NA, not available; OS, overall survival. ^†^ The status of the study has not been verified within the past 2 years.

## Data Availability

Not applicable.
